# Local sea level trends, accelerations and uncertainties over 1993–2019

**DOI:** 10.1038/s41597-020-00786-7

**Published:** 2021-01-07

**Authors:** Pierre Prandi, Benoit Meyssignac, Michaël Ablain, Giorgio Spada, Aurélien Ribes, Jérôme Benveniste

**Affiliations:** 1 CLS, Ramonville, France; 2 LEGOS/CNES/CNRS/IRD, Toulouse, France; 3 Magellium, Ramonville, France; 4 Dipartimento di Fisica e Astronomia (DIFA), Università di Bologna, Bologna, Italy; 5 CNRM/GMGEC/AMACS, Toulouse, France; 6 ESA/ESRIN, Frascati, Italy

**Keywords:** Climate-change impacts, Physical oceanography, Natural hazards

## Abstract

Satellite altimetry missions provide a quasi-global synoptic view of sea level variations over more than 25 years and provide regional sea level (SL) indicators such as trends and accelerations. Estimating realistic uncertainties on these quantities is crucial to address current climate science questions. While uncertainty estimates are available for the global mean sea level (GMSL), information is not available at local scales so far. We estimate a local satellite altimetry error budget and use it to derive local error variance-covariance matrices, and estimate confidence intervals on trends and accelerations at the 90% confidence level. Over 1993–2019, we find that the average local sea level trend uncertainty is 0.83 *mm*.*yr*^−1^ with values ranging from 0.78 to 1.22 *mm*.*yr*^−1^. For accelerations, uncertainties range from 0.057 to 0.12 *mm*.*yr*^−1^, with a mean value of 0.062. We also perform a sensitivity study to investigate a range of plausible error budgets. Local error levels, error variance-covariance matrices, SL trends and accelerations, along with corresponding uncertainties are provided.

## Abstract (summary)

**Table:** 

Measurement(s)	sea surface height
Technology Type(s)	satellite radar altimetry
Factor Type(s)	year of data collection
Sample Characteristic - Environment	sea • ocean
Sample Characteristic - Location	global

Machine-accessible metadata file describing the reported data: 10.6084/m9.figshare.13297757

## Background & Summary

Sea level is rising in response to climate change^1,2^. This rise is expected to continue over the next decades to centuries and will have far-reaching consequences for coastal societies^2,3^. Detecting, attributing and understanding the contemporary changes and trends in sea level has great importance in constraining projections of future sea level and in preparing the adaptation of coastal communities. It is also essential for climate assessments, raising public awareness, and for climate change policy development in general. In this context, quantifying precisely observational sea level uncertainties is required because uncertainties inform on the reliability of sea level observations and prevent from misinterpretations of artifacts arising from the limitations of the observing system.

Precise uncertainties are also essential for model evaluation against sea level observations^4,5^ as they enable to discriminate model-data differences that unambiguously indicate model deficiencies from those that could be due to observational errors. In that sense precise and validated uncertainties are truly indispensable for modelers when they assess their model performances against observational sea level references. Uncertainties are equally useful when comparing radar altimetry measurements with independent *in-situ* data for validation purposes.

Another application where uncertainties are essential is data assimilation. Ocean reanalyses provide dynamically consistent estimates of the physical state of the ocean over years to decades. They use available observations to constrain the dynamical evolution of an ocean model towards the observed state of the ocean. In this process the sea level observations are crucial because they provide a proxy of the ocean circulation integrated over the whole water column. The combination of observations with the ocean model involves weighting the influence of observations relative to the evolution of the model. Uncertainty estimates are needed at this stage to discriminate more certain observations from less certain ones, and give them more influence on the analysis. This is particularly important for sea level observations, as they are essential to constrain the circulation in ocean models.

Since October 1992, satellite altimetry has provided repeated precise measurements of sea level over most of the world ocean (from 82°S to 82°N), available to users on regular grids with a spatial resolution of a 1/4° on a weekly basis^6^. Measurements have been collected by radar altimeters on-board 13 different satellites that have been regularly intercompared and intercalibrated (in particular, during dedicated intercalibration phases when a satellite altimeter flies right behind its successor, a few seconds apart, to enable a close comparison of the measurements). Satellite altimeters have also been regularly validated against *in-situ* data from tide gauge records either locally in precisely monitored calibration sites like in Corsica^7^, Bass straight^8^ or California^9^ or at global scale with hundreds of tide gauge records^10^. These campaigns of satellite altimeters’ calibration and validation have given invaluable insights on the precision of the sea level measurements from space. They form the basis for the current estimations of uncertainties in sea level data.

Several studies have estimated the uncertainty in sea level data in the past. Most have focused on global mean sea level changes (hereafter noted GMSL) analysing the uncertainty on the interannual to decadal variability of the GMSL and the uncertainty on GMSL trends^10–14^. They followed two different approaches to estimate the uncertainty in sea level. A first approach relies on building a full error budget of the satellite altimetry system^11,13,14^, used to estimate the error of each component of the measurement system and propagate the associated uncertainty to the GMSL estimate accounting for the time correlation in errors (in the most recent publications). In the second approach uncertainty estimates are derived from comparisons between satellite altimetry and independent measurements from tide gauge records^10,12^. Both approaches yield similar estimates of the uncertainty in global mean sea level trends: ±0.6 *mm*.*yr* over 1993–2015 and ±0.4 *mm*.*yr* over 2002–2015 (values are given at 2*σ*, i.e. with a 95% confidence level –CL– assuming a Gaussian distribution). Comparisons between GMSL records by different groups suggest a ±2–3*mm* uncertainty in interannual variability in global mean sea level^14^. At regional level studies have focused on the impact of natural ocean variability on uncertainties rather than radar altimetry errors themselves^15–17^.

In this study we estimate, for the first time, the uncertainty in local sea level changes for all the regions of the world ocean that are sampled by satellite altimetry from errors in the measurement system. We develop an error budget analysis of the satellite altimetry system (following^13^) from which we derive the uncertainty in sea level changes at each measurement location over the period 1993–2019. We account for the time correlation in errors and we estimate at each location the temporal variance-covariance matrix of the uncertainty in local sea level. With the resulting set of variance-covariance matrices, users can derive at any location the uncertainty in any metrics associated to the local sea level changes (e.g. uncertainty in local annual sea level changes, in local sea level trends, in local sea level acceleration etc). Note that in this study we don’t account for the correlation between errors in different local sea level changes, or natural ocean variability. The estimation of the spatial correlation in sea level measurement errors is a difficult problem to tackle by itself. It is out of the scope of this paper and will be the subject of further publications.

We proceed in three steps to estimate the uncertainty in local sea level. In a first step, we identify the major sources of error that affect local sea level changes in the satellite altimetry system. We model the associated uncertainty taking into account the temporal correlation of the errors (see error budget). In a second step, we derive from this error budget the variance-covariance matrix of the errors in sea level at each location. In a third step we use the estimated variance-covariance matrices to derive the uncertainty on two metrics associated to sea level changes: the local sea level trends and accelerations over 1993–2019. To propagate the uncertainties onto the local sea level trends and accelerations, we use an optimal approach (least squares) which accounts for the information on the time correlation in errors that is included in the variance-covariance matrices. We discuss the results and conclude on the significance of the current observed regional sea level trends and accelerations over 1993–2019 (see section trend and acceleration).

## Methods

### Satellite altimetry local error budget

In the local error budget, we consider all known significant sources of errors. These include orbit determination errors, geophysical correction errors and inter-mission bias correction errors. Orbit determination errors are significant at high frequency (period <1 *yr*) and can also generate spurious drifts because of systematic effects such as drifts in the international reference frame realisation. Geophysical correction errors are significant essentially at high frequency except for the wet tropospheric correction errors which also contains low frequency constituents (period >1 yr) and the Glacial Isostatic Adjustment (GIA) correction errors which generate linear drifts. Inter-mission bias correction errors are significant at all frequencies as they generate spurious steps in sea level time series. Satellite altimetry errors are modeled as time correlated errors, drift errors or jumps^13^. Time correlated errors are characterized by their standard deviation (*σ*) and by their decorrelation time-scale (*λ*). Drifts errors are characterized by a drift magnitude (±*δ*) and jumps between successive altimetry missions are characterized by their amplitude (±Δ) timing (*t*) (see^13^ for more details). For each source of error (orbit determination, wet troposphere correction etc), for each type of error (time correlated, drift or jump), at each local point, we build the corresponding variance-covariance matrix on an annual basis. Then, for each local point, we sum the variance-covariance matrix of each source of error and of each type of errors together to construct the total local error variance-covariance matrix. Summing error covariance matrices is only valid under the assumption that errors are independent. While this is difficult to establish, the timescales and origins of the errors considered in this study differ largely so that assuming error independence is reasonable. Table 1 lists the errors considered in this study, their type, their variance and their decorrelation time-scale when relevant.

**Table 1:** Local error budget of satellite altimetry.

type	description	value	reference section
correlated	high frequency noise from orbit determination and geophysical corrections	*λ* = 1 *yr*, *σ* location dependent	section noise
correlated	low frequency noise from the wet tropospheric correction	*λ* = 10 *yrs*, σ location dependent	section wet troposhere
drift	drift errors from the orbit determination	*δ* = 0.33 *mm*.*yr*^−1^	section orbit
drift	drift errors from the GIA correction	*δ* location dependent	section GIA
jump	inter-mission TP-a/TP-b and TP-b/J1 biases	Δ = 10 *mm*	section biases
jump	inter-mission J1/J2 and J2/J3 biases	Δ = 6 *mm*	section biases

### High frequency errors from the orbit determination and the geophysical corrections

In the local error budget, the high frequency noise term accounts for short period errors in the altimeter data. These errors arise from high frequency orbit determination errors or any high frequency error in the geophysical correction including the residual tidal model errors (e.g.^18^).

For the GMSL record, high frequency noise levels were derived out of the GMSL record itself^13^ using the variance of the GMSL high frequency variability as a proxy for error levels. This is valid under the assumption that the natural GMSL variability is low and dominated by errors at high frequencies (periods shorter than 3 months) which is valid at global scale and for periods below 3 months when local SL fluctuations average out globally^11,13,14^, except when large oscillations like ENSO occur. At local level natural SL variability is the dominant signal and another high frequency noise level estimation method must be used.

The comprehensive approach to estimate the error on local sea level would be to estimate the error on the altimetry along track data and propagate this error along the processing chain on the final sea level gridded product. However along-track measurements go through such a complex filtering, cross-calibration and interpolation process to produce the final sea level grids^6,19^ that we lack today both the precise along-track error description and the precise error propagation models to estimate and propagate the error level on the final gridded product. Instead, we turn to cross-validation data to estimate the high frequency noise level on sea level grids.

At time scales shorter than 10 days, the variability in local SL is negligible compared to the noise in the altimetry measurement (e.g.^20,21^). We use the variance of differences in sea surface height (SSH), after application of all standard geophysical and instrumental corrections and empirical orbit errors correction, at crossovers separated by less than 10 days to quantify the high frequency noise between 0 and 10 days.

For time scales longer than 10 days, the natural variability in local SL is no longer negligible and we use a different estimation method. We treat separately the errors arising from the orbit determination and the correlated errors arising from the geophysical corrections. High frequency errors arising from the orbit determination are expected to sign at the harmonic frequencies of the period or the half-period of the orbit^22^. Empirical orbit determination errors are routinely estimated as part of the altimetry cross-calibration process by fitting a periodic signal at 1 and 2 cycles per revolution to SSH differences at crossovers. We use the variance of this empirical orbit error correction as the magnitude of the high frequency orbit determination induced errors on sea level estimates.

High frequency errors in geophysical corrections are essentially due to tidal and atmospheric corrections’ errors. Tidal and atmospheric corrections’ errors are expected to be correlated along track with typical decorrelation scales of 10^3^ km. In the production of the gridded altimetry products, these errors are removed during the interpolation process by the objective analysis^18^. They are also directly estimated during the production of along-track products for data assimilation purposes. We use the variance of this empirical long-wavelength error correction to quantify the local high frequency errors from geophysical corrections.

**Fig. 1:**
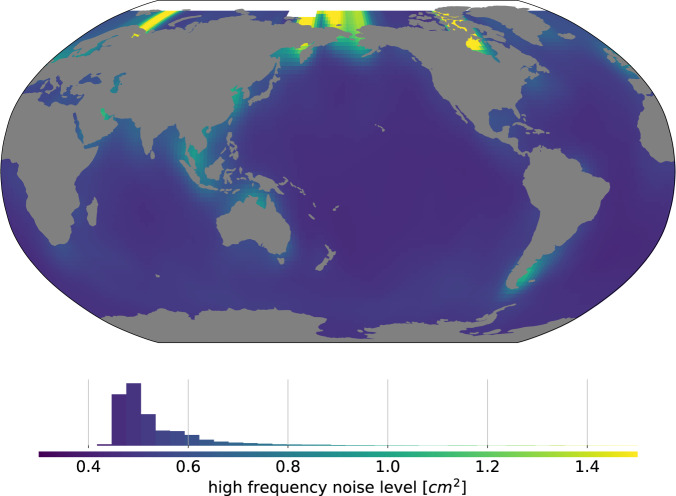
1*σ* level of the high frequency noise level in cm^2^.

Here we assume that high frequency errors from the orbit determination and the geophysical corrections are uncorrelated one from the other and they are both uncorrelated from the errors at times scales shorter than 10 days. As a result the total high frequency error is the sum of all terms, corresponding to a conservative approach, as verifying error independence is difficult and some level of cross correlation may exist. High frequency errors are also uncorrelated from one altimeter cycle (10 to 30 days) to the other. So, to derive the annual variance-covariance matrix of the total high frequency noise, for each local point, we sum the three sources of errors over each year assuming they are independent. From the resulting annual local variance-covariance matrices, we extract the local annual variances of the high frequency noise and plot them on Fig. 1. The global mean error variance level is 0.6*cm*^2^. The error variance is lower in the open ocean, and higher in coastal and high latitude regions where residual tidal and atmospheric errors tend to increase

### Low frequency errors from the wet tropospheric correction

In addition to its contribution to high frequency errors, the wet tropospheric correction exhibits low frequency errors (with time scales >1*yr*^23^). The wet tropospheric correction is derived from onboard microwave radiometer measurements of the atmosphere water content. Over the satellite altimeters’ lifetime, the radiometers’ performance degrades with age so that radiance measurement errors increase with time. This phenomenon causes low frequency errors in the wet path delay derived from the radiances and translates directly into estimates of SSH. To prevent this, microwave radiometers are regularly calibrated against reference targets such as the deep space. But, between two calibrations, some remaining low frequency errors can subsist.

Stability of the wet tropospheric correction can be assessed thanks to cross-comparisons between radiometer based solutions and atmospheric reanalyses. Several studies^23–25^ have identified long-term differences in wet tropospheric corrections computed from microwave radiometers and from atmospheric reanalyses (e.g. ERA-interim reanalyses^26^). These studies report differences in the wet tropospheric correction for GMSL in the range of ±0.2–0.3 *mm*/*yr* for periods of 5 to 10 years. They also show that the differences in wet tropospheric corrections are generally latitude dependent, with largest differences in the tropical band, where the wet tropospheric correction is large and lowest differences at high latitudes, where the wet tropospheric correction is low.

**Fig. 2:**
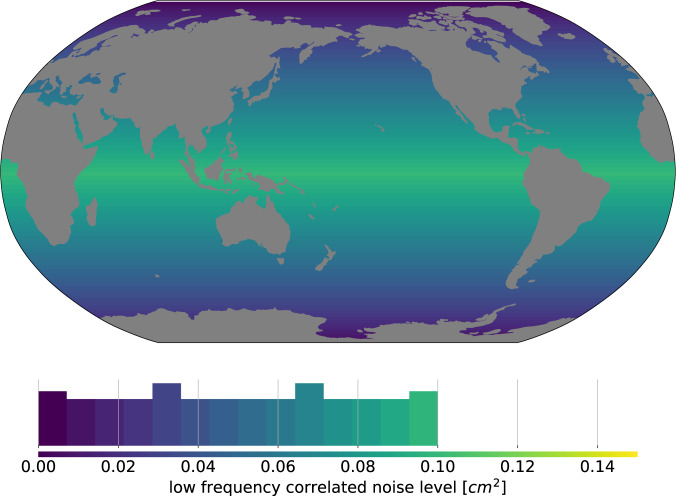
1*σ* level of the low frequency correlated error related to wet tropospheric correction errors in cm^2^.

Here we adopt a conservative approach and we model the local error in wet tropospheric correction by a correlated noise characterised by a decorrelation time scale of 10 years. The noise variance level is linearly scaled with the latitude and chosen such that the global variance level fits the variance level chosen in^13^. The corresponding variance levels are represented on Fig. 2.

### Drift errors from the orbit determination

Historically, drifts in the orbit solutions are important contributors to the local sea level uncertainties (see for example^6^). Drifts in orbit solutions are essentially due to drifts in the international terrestrial reference frame realisation in which orbit solutions are estimated and also to drifts in the time-variable gravity field used to estimate the orbit solutions. Recently^27^ and^28^ showed that orbit solutions computed from different processing centers are converging towards a better agreement with differences that do not exceed 1 mm/yr locally. This is confirmed by the comparisons between altimetry products using two orbit solutions (POE-D and POE-E) which exhibits trend differences below 1 mm/yr locally.

However, there is poor agreement between orbital solutions on the geographical pattern of the drifts. The difference between the current POE-D and POE-E orbit solutions shows an east/west pattern but this pattern changes drastically with each new version of the POE products (see for example^14,29^). The reasons for this are still under investigation.

Because of the limited understanding of the regional pattern in orbital drift errors, we choose not to specify any pattern for the orbital determination drift errors. We adopt a conservative approach and prescribe a uniform orbit determination drift error over the globe using the highest level of error stated in^27^. We prescribe a uniform orbital drift error of ±0.33 *mm*/*yr*^−1^ which is the 1*σ* level with respect to the maximum error level of 1 *mm*/*yr* stated in^27^.

### Drift errors from the GIA correction

To measure the current climate related contribution to regional sea level rise, sea level records should be corrected for glacial isostatic adjustment (GIA) effect using a model (e.g.^30^). For global mean sea level, this correction adds an extra 0.3 *mm*.*yr*^−1^ to the observed trend, with an uncertainty of ±0.05 *mm*.*yr*^−1^ At the regional scale, the GIA correction can reach up to 1.5 *mm*/*yr*^−1^, and associated errors are also higher than at global scale. Here the GIA contribution to the present day rate of geocentric sea level^31^ change was removed and its uncertainty evaluated regionally by considering an ensemble of 27 solutions of the Sea Level Equation (SLE). The SLE has been solved on a global grid to harmonic degree 256 according to the classical theory of ^32^, but taking into account the Earth rotation, using the pseudo-spectral method and the iterative approach described by^33^. Earth rotation has been modeled adopting the “traditional rotation theory” (see^33^ and references therein). In the 27 SLE runs, we have kept the same history of deglaciation of the late-pleistocene ice sheets: the history of deglaciation from the ICE-5G(VM2) model^30^. In each of the 27 runs, the viscosity of the shallow upper mantle (SU), transition zone (TZ) and lower mantle (LM) is varied in the range 0.2 to 0.8 10^21^*Pa.s* (SU and TZ) and 2.0 to 3.4 10^21^*Pa.s* (LM), respectively. These intervals include the nominal VM2 viscosity profile as their central value. In all the runs the lithospheric thickness has been kept fixed to 90 km (we have verified that it has minor effects on the global pattern of sea level rise). We also tested other models like e.g. ICE-6G C (VM5a)^34^ but they lead to results that are not significantly different (not shown).

**Fig. 3:**
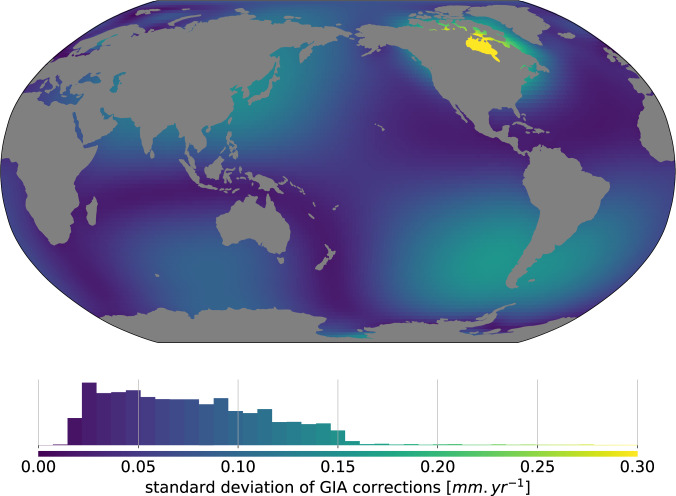
Standard deviation of GIA corrections from an ensemble of 26 members, in *mm*.*yr*^−1^.

The resulting uncertainty (given at 1 *σ* level) is shown on Fig. 3. The GIA induced error is generally a low contribution with respect to the orbital drift. In some areas however, the error on this correction can reach ±0.3 *mm*.*yr*^−1^, for example in the Hudson Bay area.

### Inter-mission bias errors

In general, local sea level is estimated from an objective analysis of all altimetry data collected by all available radar instruments flying on different platforms, over different orbits, and different time periods. For example, CMEMS products^35^ use all available altimetry measurements and C3S product^6^ use at each time step the measurement from two different missions (to remain homogeneous along the record). Measurements from different satellites are combined through an optimal interpolation scheme after precise cross-calibration^18^ which ensures that secondary missions are tied to the reference mission (namely TOPEX, Jason 1,2 and 3).

In the gridded products, switches from one reference mission to another are critical for the long term record stability^36^. Over the 1993–2019 period, such switches occur four times: from TOPEX-A to TOPEX-B, from TOPEX-B to Jason-1, from Jason-1 to Jason-2 and from Jason-2 to Jason-3. To ensure the long term stability of the record, inter-mission biases are estimated for each switch during an inter-calibration phase when a satellite reference mission flyes right behind its successor a few seconds apart (except for the switch from TOPEX-A to TOPEX-B because both instruments were on board the same spacecraft). The accuracy of the inter-mission bias estimate depends on the length of the inter-calibration phase and noise levels of the two missions involved.

Regarding global average biases^13^, estimate that uncertainties on inter-mission biases amount to ±2 *mm* for the TOPEX-A/TOPEX-B transition, and to ±0.5 *mm* for the TOPEX-B/Jason-1 and Jason-1/Jason-2 transitions. Uncertainty on the inter-mission bias is higher for the TOPEX-A to TOPEX-B transition because of the lack of inter-calibration phase^36^.

Regarding the regional distribution of inter-mission bias error, the standard deviation of SSH differences during the TOPEX-B/Jason-1, Jason-1/Jason-2 and Jason-2/Jason-3 inter-calibration phases suggests that the regional bias uncertainty is around ±10 *mm* for the TOPEX-B/Jason-1 transition and ±6 *mm* for the Jason-1/Jason-2 and Jason-2/Jason-3 transitions (at the 1*σ* level). This is about one order of magnitude larger than the global numbers from^13^. This is because there are large scale spatial correlations in the bias uncertainty (not shown) that compensate when averaged at global scale. These large scale spatial correlations in the bias uncertainty change with time and the reasons for this change is not elucidated yet. Here we adopt a conservative approach and take a uniform bias uncertainty at ±10 *mm* for the TOPEX-B/Jason-1 transition and ±6 *mm* for the Jason-1/Jason-2 and Jason-2/Jason-3 transitions (at the 1*σ* level). In the absence of data to analyse the transition between TOPEX-A and TOPEX-B, we assume the same bias uncertainty for the TOPEX-A/TOPEX-B transition as for the TOPEX-B/Jason-1 transition.

### Uncertainties in local trends and accelerations

In this section we use the variance-covariance matrices estimated in the previous sections to derive the uncertainty on the local sea level trends and accelerations over the period 1993–2019. For each local point, we sum the variance-covariance matrices associated to each source of uncertainty and we estimate the uncertainties onto the local sea level trends accelerations with an optimal approach (extended least squares) which accounts for the information on the time correlation in errors included in the variance-covariance matrices.

### Sea surface height data

To estimate the local sea level trends and the local sea level acceleration we choose to use the SLA grids derived from satellite altimetry measurements from the Copernicus Climate Change Service (C3S)^6^. This dataset focuses on long-term stability for climate applications and provides an homogeneous processing from 1993 to present. Daily SLA maps at 1/4° spatial resolution are available from https://cds.climate.copernicus.eu. The time span considered here is limited to 1993 to 2019 to consider only complete years. C3S sea level grids are corrected for a time-varying TOPEX-A drift following^37^ and for local geocentric SL changes due to GIA. Here We focus on large scale sea level patterns (both in space and time) which respond to climate fluctuations. Individual meso-scale eddies can generate substantial variability in sea level and mask the underlying climate fluctuations. To remove this noise from eddies we perform a spatio-temporal smoothing of the original grids. A 3° wide gaussian spatial filter is applied to remove high frequency spatial features and yearly averages are computed from the daily grids. After filtering, we sub-sample the grids to a 2° resolution. This filtering is consistent with previous works (e.g.^5,38^). Grid points where data coverage is incomplete are discarded from the analysis (leading to the removal of the seasonally ice covered Arctic ocean and Southern Ocean from our study).

### Trend and acceleration uncertainty estimate

We address the estimation local SL trends, accelerations and associated uncertainties in local sea level. To do so, we use a first or second order polynomial as a predictor of the local sea level changes of the form $\widehat{y}=\sum {a}_{i}{x}^{i}$. Acceleration is estimated as twice the quadratic coefficient. At a given location and for a second order fit, considering the local sea level record has *n* observations, let *X* be an *n* by 3 predictor where the first column contains only ones (representing the constant term), the second column contains the time vector (representing the linear term) and the third column contains the square of the time vector (representing the squared term). Let *y* be an *n* by 1 vector of independent observations of the local sea level changes. Let *ε* be an *n* by 1 vector of disturbances (local sea level signal that is non-linear and non-quadratic with respect to time) and errors. Let *β* be the 3 by 1 vector of unknown parameters that we want to estimate. Our linear regression model for the estimation of the local sea level trend and acceleration will thus be

1 $$y=X\beta +\varepsilon $$

with

2 $$\varepsilon  \sim N(0,\Sigma )$$

where ∑ is the sum of the variance-covariance matrices of the observation errors (estimated in the previous section).

The most common method to estimate the local sea level trend and acceleration from Eq. 1 is to use an ordinary least square approach^39^. But in this approach, the estimate of the uncertainty in trend and acceleration does not account for the correlation in errors^39^. To cope with this issue, we turn to an extended least square approach, following^13^ and previous authors^11,40^ (and IPCC AR5, Box 2.2 and Supplementary Material)^41^. In the extended least square approach, the estimator is the same as the ordinary least square estimator (it is also unbiased like in the ordinary least square approach), but its distribution is revised to account for ∑, leading to the expression:

$$\widehat{\beta }=N(\beta ,{({X}^{t}X)}^{-1}{X}^{t}\Sigma X{({X}^{t}X)}^{-1})$$

**Fig. 4:**
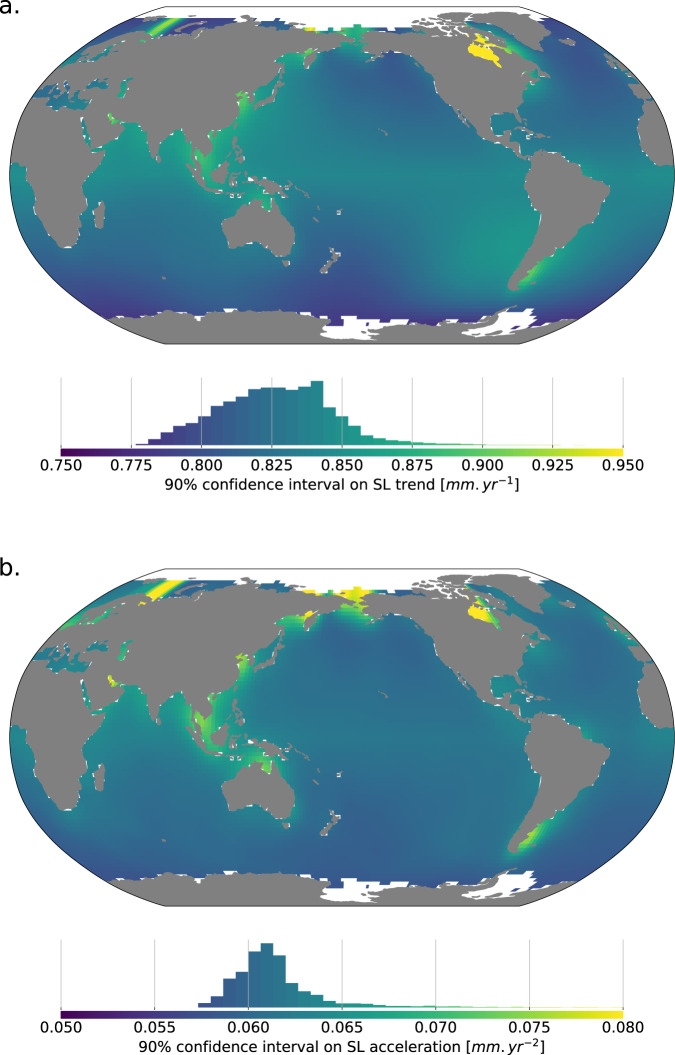
Map of 90% confidence intervals half-width on (**a**) sea level trends (in *mm*/*yr*) and (**b**) sea level acceleration (in *mm*/*yr*^2^) observed from altimetry measurements.

For each local grid point of the SSH maps, we fill the error variance-covariance matrix following the error budget terms described in section error budget, and perform the estimation of $\widehat{\beta }$. The resulting uncertainties on local sea level trends and accelerations are converted to 90% confidence intervals following quantiles of a two-tailed Student law, and are shown on Fig. 4.

The uncertainty in local sea level trend ranges from 0.78 to 1.22 *mm*.*yr*^−1^ with a mean value of 0.83 *mm*.*yr*^−1^. Locally, the uncertainties on sea level trends are found to be 2 to 4 times higher than the uncertainty on the GMSL trend. The average local SL trend uncertainty is about twice the estimate GMSL trend uncertainty from^13^.

The uncertainty in local sea level acceleration ranges from 0.057 to 0.12 *mm*.*yr*^−2^ with a mean value of 0.062 *mm*.*yr*^−2^. Uncertainties on local SL accelerations are 1 to 2 times higher than the uncertainty on the GMSL acceleration. The average local SL acceleration uncertainty is comparable to the GMSL acceleration uncertainty^13^. This is a consequence of different error budget modelling choices. Low frequency orbit errors are modelled here as a drift and have no impact on acceleration uncertainty. They are modeled as a drift and low frequency correlated error and therefore contribute to acceleration uncertainty in^13^

Spatial patterns of uncertainty in SL trends and accelerations are similar. For both trends and accelerations, we find that uncertainties are generally higher at the coast as a result of higher levels of the high frequency noise at the coast (especially the noise associated to the geophysical corrections such as tides and dynamical atmospheric correction). Large scale patterns are dominate by GIA error for uncertainty on trends, and low frequency wet tropospheric correction error for uncertainty on accelerations.

### Significant local sea level trends and accelerations

**Fig. 5:**
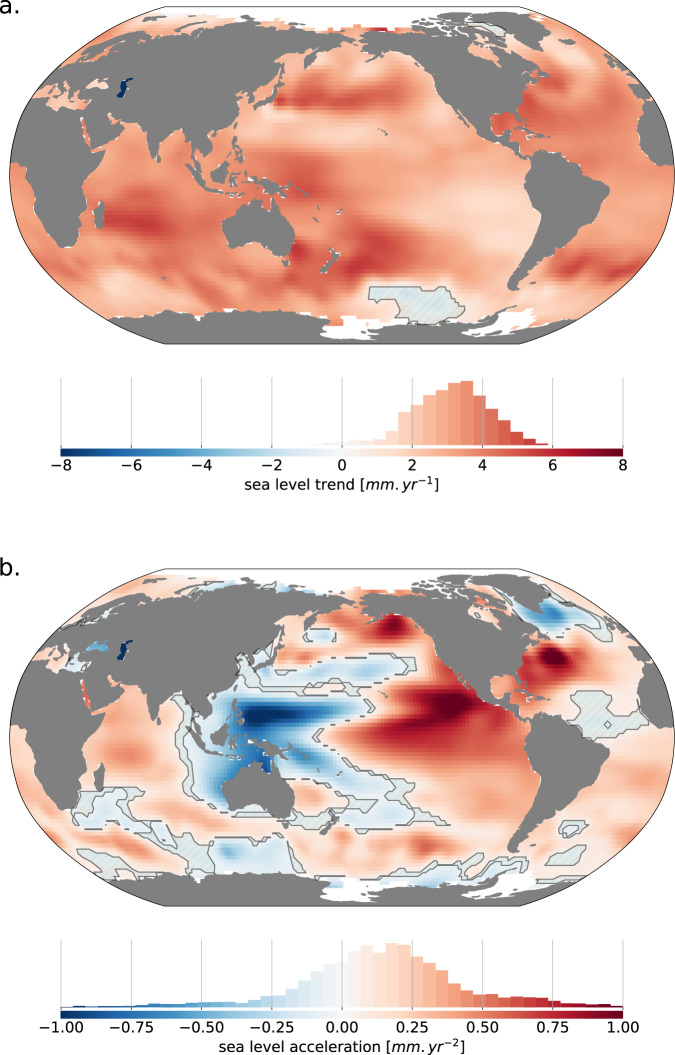
Map of (**a**) sea level trends (in *mm*/*yr*) and (**b**) sea level accelerations (in *mm*/*yr*^2^) over 1993–2018. Hatched areas indicate regions where the sea level trend or acceleration are not significant at the 90% confidence level because of instrumental errors.

To identify the regions where the measured sea level trends are significant above the instrumental and post processing noise estimated in this study, we compare the estimate of the trend uncertainty (at the 90% confidence level) with the observed sea level trends. We find that 98% of the ocean surface experiences a significant sea level rise (see Fig. 5). The few regions where sea level trends are not significant, are located in the Southern Ocean, Baffin Bay and in the north Atlantic Ocean, south of Iceland. In all areas where sea level is falling (see Fig. 5) the rate of sea level fall is not significant at the 90% confidence level, except in the Caspian Sea.

The pattern of sea level acceleration is very different from the pattern of sea level trends. It is much less uniform (see Fig. 5) with an east/west dipole in the Pacific, a north/south dipole in the southern ocean and in the north Atlantic. Most of the regions of the world (85%) show a significant sea level acceleration or deceleration, above the instrumental and post processing noise estimated in this study (at the 90% confidence level). The areas where the sea level acceleration or deceleration are not significant are the regions where the acceleration is close to 0. These areas are essentially located along the front line between the positive and negative center of each dipole.

## Data Records

Local error variance-covariance matrices, along with local sea level trends, accelerations and associated uncertainties are distributed as a single netCDF file. This file is freely available online at 10.17882/74862^42^. The dataset also contains annual mean sea level grids and error levels needed to reproduce the results of this paper.

## Technical Validation

### Sensitivity analysis

**Fig. 6:**
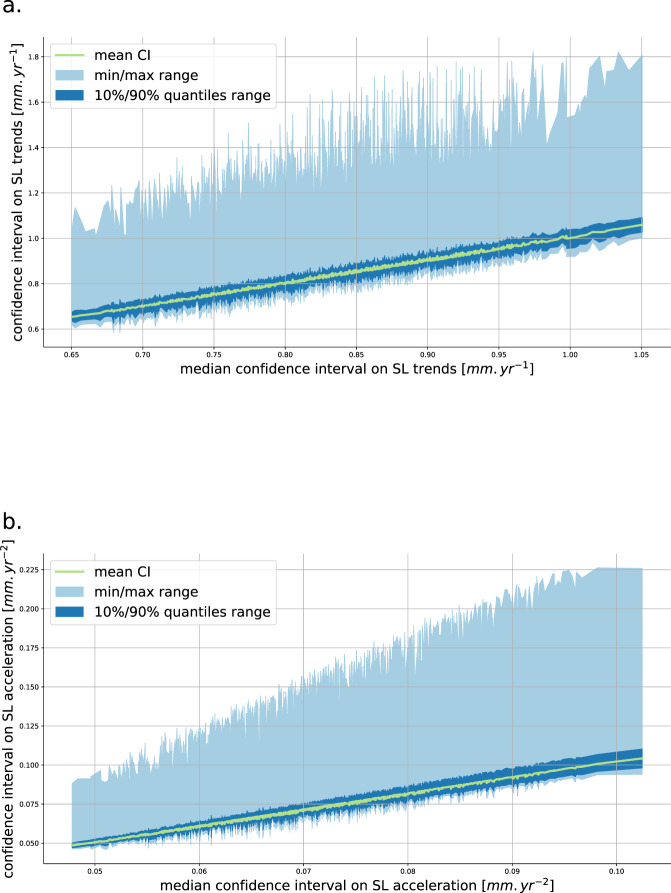
Effect of changes in the error budget described in Table 2 on the confidence intervals on local sea level trends (**a**) and accelerations (**b**). The [minimum;maximum] and [10%, 90%] quantiles are shown for each sample, the mean confidence interval is overlaid in green.

Uncertainties estimated in this study are dependent on the error budget developed in section error budget. This error budget is based on our current best knowledge of the altimetry measurement system errors. As the altimetry record increases in length with new altimeter missions, the knowledge of the altimetry measurement also increases and the description of the errors improves. Consequently, the error budget description is also expected to change and improve in the future leading to new estimates of the uncertainties in local sea level trends and accelerations. To asses to which extent the uncertainties in local sea level trends and acceleration may change in the future we evaluate the sensitivity of the uncertainties to changes in the error budget. We perform 1000 experiments where each error source of the error budget is perturbed in amplitude and decorrelation time scale when relevant. Perturbations are drawn from a range of plausible values (see Table 2) using a uniform distribution. From each perturbation experiment we estimate the total range, the mean and the median of local uncertainties in sea level trends and sea level accelerations. The results from this sensitivity analysis are ranked from the most optimistic to the most pessimistic error budget and plotted on Fig. 6.

**Table 2:** Ranges of parameter values used for the sensitivity analysis.

source of uncertainty	parameter nominal value	sensitivity test range
*high frequency noise	*σ* (location dependent, see section noise)	[0.5*σ*;2*σ*]
	*λ* = 1 *yr*	[0.1;1] *yrs*
*wet tropospheric error	*σ* (location dependent, see section wet troposphere)	[0.5*σ*;2*σ*]
	*λ* = 10 *yr*	[5;15] *yrs*
orbit determination drift	*δ* = 033 *mm*	[0.2;0.4]*mm*/*yr*
TP-a/TP-b and TP-b/J1 bias	*σ* = 10 *mm*	[7;13] *mm*
J1/J2 and J2/J3 bias	*σ* = 6 *mm*	[3;9] *mm*

Uncertainties in the sea level trends are not very sensitive to changes in the error budget specifications. From the most optimistic error budget to the most pessimistic one, in which amplitudes of the errors are multiplied by a factor of 2 to 4, the average local sea level trend uncertainty increases from 0.65 to 1.05 *mm*.*yr* and the 10–90% quantiles of the distribution increases from [0.63; 0.68] to [1.03; 1.1] In the worst case, uncertainty in local SL trend is below 1.9 *mm*/*yr*, and reaches this value only at a few extreme points. For all samples, 80% of the distribution of local SL trend uncertainties remain below 0.92 *mm*/*yr* and the ratio of the ocean area that experiences significant sea level rise remains greater than 97%.

Uncertainties on sea level accelerations are slightly more sensitive to changes in the error budget (see 6, panel b). The mean uncertainty goes from 0.049 to 0.10 *mm*.*yr*^−2^, while 10–90% intervals increase from [0.047; 0.050] to [0.1; 0.11]. The largest acceleration uncertainty across all perturbations is 0.23 *mm*.*yr*^−1^. The ratio of the ocean area that experiences significant sea level acceleration ranges from 88% (most optimistic) to 75% (most pessimistic).

## Usage Notes

The dataset is made available as a self-describing NetCDF file (UCAR/Unidata, 10.5065/D6H70CW6).

## Data Availability

All the code needed to reproduce this study is released under the GNU GPL v3 license. The code is available at https://github.com/pierre-prandi/rsl.
